# Zinc and copper levels in low birth weight deliveries in Medani Hospital, Sudan

**DOI:** 10.1186/1756-0500-7-386

**Published:** 2014-06-24

**Authors:** Rihab M Abass, Hamdan Z Hamdan, Elhassan M Elhassan, Sumia Z Hamdan, Naji I Ali, Ishag Adam

**Affiliations:** 1 Sudan Academy of Science, Khartoum, Sudan; 2 Faculty of Medicine, Al-Neelain University, P.O. Box 12702, Khartoum, Sudan; 3 Faculty of Medicine, University of Gezira, Wad Medani, Sudan; 4 Sudan Atomic Energy Commission, Khartoum, Sudan; 5 Faculty of Medicine, University of Khartoum, P.O. Box 102, Khartoum, Sudan

**Keywords:** Zinc, Copper, Low birth weight, Sudan

## Abstract

**Background:**

Low birth weight (LBW) is a worldwide health problem, especially in developing countries. We conducted a case–control study at Medani Hospital, Sudan. Cases were women who delivered a LBW (<2500 g) newborn and consecutive women who delivered a normal weight (>2500 g) newborn were controls. Questionnaires were used to collect clinical data. Zinc and copper levels were measured by an atomic absorption spectrophotometer.

**Findings:**

The two groups (50 in each arm) were well matched in their basic characteristics. Median (25–75th interquartile range) maternal zinc (62.9 [36.3–96.8] vs. 96.2 [84.6–125.7] μg/dl; *P* <0.001) and copper (81.6 [23.7–167.5] vs. 139.8 [31.9–186.2] μg/dl*; P* = 0.04) levels were significantly lower in cases than in controls. Cord copper levels in cases were significantly lower than those in controls (108 [55.1–157.9] vs. 147.5 [84.5–185.2] μg/dl; *P =* 0.02). There were significant direct correlations between birth weight and maternal copper levels and maternal and cord zinc levels.

**Conclusions:**

Maternal zinc and copper levels, as well as cord copper levels, are lower in LBW newborns than in those with normal weight.

## Findings

Low birth weight (LBW) is a major health problem in developing countries, especially in sub-Saharan Africa, including Sudan [1,2]. Birth weight is the main predictor of infant morbidity, survival, and health status [3]. LBW (<2500 g) has many adverse outcomes, such as increased susceptibility to adulthood diseases (e.g., coronary heart disease, diabetes mellitus, hypertension, and obstructive lung disease) [4,5], and it is the leading cause of perinatal and infant mortality [6,7].

Zinc and copper are important trace elements that are needed for optimum human growth and development [8]. Zinc and copper are cofactors in many antioxidants enzymes that protect the cell against free radicals [9,10]. Zinc is involved in normal metabolic and physiological processes that control cell growth [11]. Zinc requirements are increased during pregnancy [12]. Low levels of zinc and copper are independently associated with a risk of LBW neonates [13-15].

LBW is a common health problem in different regions of Sudan and maternal anemia is the main risk factor for LBW [2,16-18]. A high rate of zinc and copper deficiency has been reported among pregnant Sudanese women, regardless of their age and parity [19]. There are few published data on maternal and cord levels of zinc, copper, and LBW deliveries, with contradictory results [14,15,20-22] Therefore, these elements need to be investigated in LBW neonates to provide health planners and care-providers with fundamental data necessary for appropriate intervention. The current study was conducted at Medani Hospital to investigate maternal and cord blood levels of zinc and copper in LBW newborns.

## Methods

A case–control study was conducted at Medani Maternity Hospital in Central Sudan during May to October 2010. Medani is the capital of Al Gezira state, which is the second largest state in Sudan. The state of Al Gezira has a surface area of 26,0752 km^2^ and 4.133.048 inhabitants, which represent 11.1% of the total population of Sudan. According to World Bank data, the gross national income per capita in Sudan is $1500. Sudan is a developing country with a low middle-income level and 46.5% of its population is below the poverty line [23]. Medani Maternity Hospital is a tertiary hospital in Al Gezira state, and this receives referral cases from different medical centers and patients who live close to the hospital. After signing an informed consent form, women with a singleton neonate were approached to participate in this study. Cases were women who delivered a term newborn weighing ≤2499 g. For every case, a subsequent woman who delivered a term newborn weighing ≥2500 g by normal vaginal delivery acted as a control. Cases and controls were women who used iron and folic acid supplement only and no any other supplements. Sociodemographic data were gathered using a structured questionnaire for each woman. The obstetrical history, including information on the first day of the last menstrual period before the index pregnancy and on the date of previous pregnancy outcomes (delivery and miscarriage), was gathered. The inter-pregnancy interval was defined as the time between the woman’s previous delivery, miscarriage and the first day of the last menstrual period for the index pregnancy. The date of the last normal menstrual period was used to determine gestational age. However, when a discrepancy between gestational age determined in this way and gestational age calculated from ultrasound scanning was greater than 2 weeks, the ultrasound estimate was preferred. All women with diabetes mellitus, a hypertensive disorder of pregnancy, those who developed obstetrical complications, such as gestational diabetes and antepartum hemorrhage, or those who suffered from any medical illness were excluded. Maternal weight, height, and body mass index, which was calculated as weight in kilograms divided by height in meters squared, were obtained. Newborns were immediately weighed with an electronic digital scale to the nearest 50 g.

Maternal hemoglobin was measured using the HemoCue hemoglobinometer (HemoCue AB, Angelhom, Sweden).

Maternal venous and cord blood was immediately collected after birth from each woman and allowed to clot in plain tubes. Serum was stored at -20°C until later analysis in a laboratory in Khartoum for measurement of serum zinc and copper concentrations. Zinc and copper concentrations were measured by an atomic absorption spectrophotometer (SOLAAR, Atomic Absorption Spectrophotometer, Thermo Electron, Cambridge, UK).

### Statistical analysis

Data were entered in a computer using SPSS for Windows version 16.0 and double-checked before analyses. Data were checked for normality. Data are shown as mean (SD) when normally distributed and median (25–75th interquartile range) when not normally distributed. To compare mean variables, the Student’s *t* test was used for normally distributed data and the Mann–Whitney *U* test was used if data were not normally distributed. Pearson’s correlation was used to assess the association of zinc and copper with clinical, biochemical, and obstetrical data, and the correlation between these clinical and biochemical data. *P* < 0.05 was considered significant.

### Ethics

This study received ethical clearance from the Research Board at the Faculty of Medicine, University of Khartoum, Sudan.

## Results

**Figure 1:**
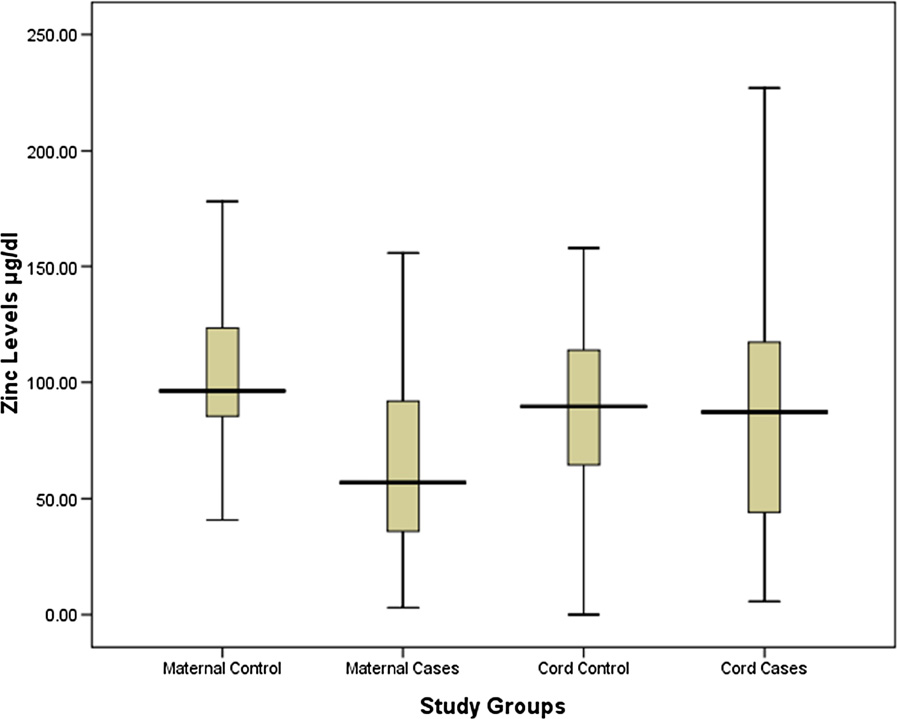
Maternal and cord levels of zinc in LBW cases and controls in Medani Hospital, Sudan.

**Figure 2:**
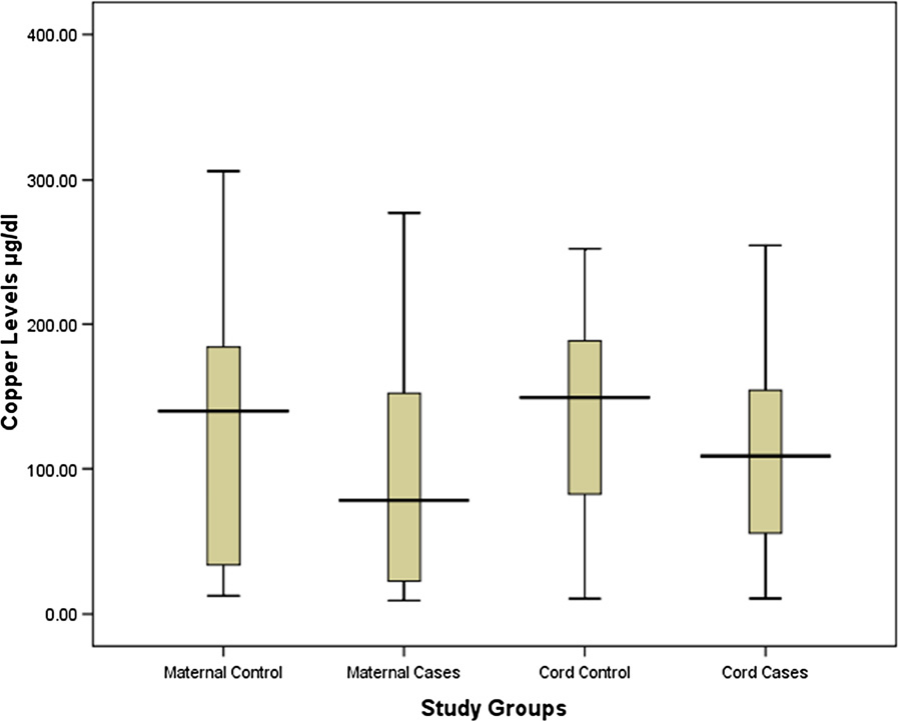
Maternal and cord levels of copper in LBW cases and controls in Medani Hospital, Sudan.

The two groups (50 in each arm) were well-matched in their basic characteristics (Table 1). Maternal serum zinc (62.9 [36.3–96.8] vs. 96.2 [84.6–125.7] μg/dl *P* < 0.001) and copper (81.6 [23.7–167.5] vs. 139.8 [31.9–186.2] μg/dl; *P* = 0.04) levels were significantly lower in cases with LBW deliveries than in controls. Similarly, cord copper levels were significantly lower in cases with LBW deliveries than in controls (108 [55.1–157.9] vs. 147.5 [84.5–185.2] μg/dl; *P* = 0.02). Cord zinc levels were lower in cases than in controls (87.1 [43.3–118.1] vs. 92.2 [65.0–114.5] μg/dl; *P* = 0.49), but this did not reach statistical significance (Table 2, Figures 1 and 2).

**Table 1:** Sociodemographic, clinical, and biochemical variables of women with low birth weight and controls

**Variable**	**Controls (*n*** **= 50)**	**Cases (*n*** **= 50)**	***P *** **value**
Age, years	27.54 (5.06)	27.5 (6.3)	0.98
Parity	2.04 (1.5)	2.1(2.3)	0.80
Gestation age, weeks	38.1(1.2)	37.8(1.9)	0.43
Inter-pregnancy interval, months	22.5(16.7)	16.6(17.7)	0.09
Weight, kg	65.02 (14.3)	59.9 (12.7)	0.06
Body mass index, kg/m^2^	25.4(3.9)	23.9(4.5)	0.08
Hemoglobin, g/dl	11.1 (1.4)	10.8(1.4)	0.38

Values are mean (SD).

**Table 2:** Zinc and copper levels in maternal and cord blood

**Variable**	**Cases (*n*** **= 50)**	**Control (*n*** **= 50)**	***P *–value**
Maternal zinc	62.9 (36.3 – 96.8)	96.2 (84.6 – 125.7)	<0.001
Cord zinc	87.1(43.3 – 118.1)	92.2 (65.0 – 114.5)	0.49
*P* –value	0.15	0.12	
Maternal copper	81.6 (23.7 – 167.5)	139.8 (31.9 – 186.2)	0.04
Cord copper	108 (55.1 – 157.9)	147.5 (84.5 – 185.2)	0.02
*P* –value	0.16	0.46	

There were significant direct correlations between birth weight and maternal zinc levels (*r* = 0.27, *P* = 0.005), and between birth weight and maternal copper levels (*r* = 0.19, *P* = 0.05). Similarly, cord zinc levels were positively correlated with maternal zinc levels (*r* = 0.29, *P* = 0.003) and birth weight (*r* = 0.35, *P* = 0.01, Table 3).

**Table 3:** Correlations among zinc, copper, and birth weight

**Variables**	**Maternal zinc**		**Maternal copper**		**Cord zinc**		**Cord copper**	
	***r***	***P***	***r***	***P***	***r***	***P***	***r***	***P***
Birth weight	0.27	0.005	0.19	0.05	0.35	0.01	0.1	0.23
Maternal zinc			0.39	< 0.001	0.29	0.003	0.13	0.16
Maternal copper	0.39	< 0.001			0.18	0.07	0.04	0.63
Cord zinc	0.29	0.003					0.004	0.96

## Discussion

The main findings of the current study were that maternal zinc and copper levels were lower and cord copper levels were lower in LBW deliveries compared with controls. This could explain recent findings at the same hospital, where anemic women were at nine times the risk of LBW delivery [2] and 45% of antenatal attendees had zinc deficiency [19]. The finding of a low zinc level in LBW deliveries is in agreement with recent reports from developing countries, such as Tanzania [14] and India [15]. Maternal zinc deficiency can lead to an adverse pregnancy outcome, such as intrauterine growth retardation [24,25], pregnancy-induced hypertension, and LBW deliveries [26,27]. However, previous studies did not show any significant difference between birth weight in mothers with zinc deficiency [21], and birth weight was not correlated with maternal blood levels of zinc [20]. Badakhsh et al. [22] observed no association between LBW deliveries and low maternal zinc status. Likewise, recently, Gebremedhin et al. found no effect of prenatal zinc deficiency on newborn birth weight [28].

In the current study, as well as in a previous report, LBW newborns had higher cord zinc levels than their mothers [29]. However, Srivastava et al. [30] reported that maternal zinc levels are higher than cord zinc levels. The growing fetus is likely to store zinc in an escalating manner as gestational age advances [31]. There might be an active mechanism for transporting zinc across the placenta from the maternal side toward the fetus [32]. A clinical trial of pregnant women who were supplemented daily with 25 mg of zinc showed a positive outcome in terms of birth weight and head circumference compared with the placebo group [33]. Maternal zinc deficiency in late pregnancy could be a determinant for newborn birth weight [34]. Recently, a study reported that LBW neonates with zinc deficiency are prone to lose more zinc in their early infancy [35].

In the current study, significantly lower copper levels were observed in maternal and cord serum in LBW cases than in controls. During pregnancy, maternal copper increases as gestational age advances. This finding could be explained by an increase in synthesis of ceruloplasmin, a copper binding protein, in response to high levels of estrogen during pregnancy [36]. This ensures the availability of copper, as fetal and maternal demand increases [37]. A growing body of evidence has linked low maternal copper levels and LBW [13,37]. Copper deficiency during the prenatal, and even the early postnatal periods, can lead to defective energy production, altered bone structure, dysgenesis of blood vessels, and delayed lung development. Unfortunately, these changes might not subside, even with copper supplementation to deficient neonates [37].

In our study, in the case and control groups, copper levels were higher in cord blood than in maternal blood. This finding could be the cause of intrauterine growth restriction, as reported recently [38]. Copper concentrations in maternal and cord blood in cases and the control group were higher than zinc concentrations. High copper concentrations lead to competition with zinc for metallothionein binding sites, and thus decrease zinc availability [39]. In this study, there was a significant, strong, positive correlation between birth weight and maternal and cord zinc concentrations, as found in a previous study [40]. A short inter-pregnancy interval is a risk factor for developing LBW [41-43]. Nevertheless, this factor was not significant in our study.

Maternal anemia is a contributing factor for LBW deliveries [2]. Zinc deficiency has been identified as a risk factor for maternal anemia [44]. Serum copper is not a predictor for maternal anemia, but significant copper deficiency has been observed in pregnant women with anemia [19]. Moreover, in non-pregnant adolescents, there is a positive correlation between hemoglobin levels and serum zinc and copper levels [45]. Deficiency in zinc and copper, and anemia might be interacting factors in the etiology of LBW because all of them can be involved maternal malnutrition.

This study has several limitations. First, the dietary content of zinc and copper was not quantified in this study. Second, measuring serum copper levels is less sensitive for detecting marginal copper deficiency, and this is similar for serum zinc levels. Third, this study was not designed to assess the differences in concentration of these elements in different trimesters. Therefore, further studies are required to determine these issues.

## Competing interests

The authors declare that they have no competing interests.

## Authors’ contributions

RMA, HZH, NIA, and SZH carried out the study and participated in the statistical analysis and procedures. IA and EME coordinated and participated in the design of the study, statistical analysis, and drafting of the manuscript. All the authors read and approved the final version.
